# Refractive lens exchange with trifocal intraocular lens after radial keratotomy

**DOI:** 10.3205/oc000259

**Published:** 2025-11-21

**Authors:** Şefik Can İpek, Seher Köksaldı

**Affiliations:** 1 Department of Ophthalmology, Dunyagoz Suadiye Private Hospital, Istanbul, Turkey; 2 Department of Ophthalmology, Agri Ibrahim Cecen University, Agri, Turkey

**Keywords:** cataract surgery, intraocular lens calculation formulas, presbyopia correcting, radial keratotomy, trifocal intraocular lens

## Abstract

**Objective:**

To report a case with a history of previous radial keratotomy who underwent refractive lens exchange with trifocal intraocular lenses.

**Methods:**

Case report

**Results:**

A 69-year-old man underwent bilateral RK to correct myopia in both eyes elsewhere 40 years ago. He was admitted to our hospital due to progressive deterioration of vision. The uncorrected distance visual acuity in both eyes was 20/40, and the uncorrected near visual acuity (40 cm) was J10. On slit-lamp examination, mild cataract and four radial keratotomy incisions were found. The patient underwent bilateral refractive lens exchange with trifocal intraocular lenses one-week interval. We were unable to obtain the preoperative data or details of the patient’s prior surgeries. Calculations were run with the American Society of Cataract and Refractive Surgery calculator using the Barret True K formula. Postoperative follow-up was continued for approximately six months. No perioperative complications were noted.

**Conclusions:**

Six months after the surgery, the refractive outcomes for both eyes had stabilized, and no other complications had occurred. The patient was satisfied with the result.

## Introduction

Radial keratotomy (RK) was the predominant treatment for myopia in the late 1970s to 1980s before the development of excimer laser surgery. To correct myopia, the approach included creating 4 to 32 radial incisions to flatten the central cornea [1].

Calculating intraocular lens (IOL) power for eyes post-radial keratotomy poses some challenges. These challenges arise from difficulties in accurately determining the true corneal refractive power, which is due to the anterior and posterior corneal irregularities induced by the RK incisions as well as difficulties in accurately predicting the effective lens position. Also using standard IOL calculation methods causes the hyperopic shift that occurs over time [1], [2]. Furthermore, the diminished strength of the cornea increases the risk of radial corneal incision dehiscence during intra- or postoperative stages, resulting in a prolonged duration until refraction stability [3].

Most surgeons tend to avoid refractive surgery in patients with RK and prefer monofocal IOLs due to existing difficulties and unpredictable outcomes. Few cases of multifocal or extended depth of focus lenses (EDOF) after RK surgery have been reported in the literature [4], [5], [6]. Herein, we report a case with hypermetropic shift and presbyopia after previous RK in which we performed refractive lens exchange and implanted trifocal IOLs. 

## Case description

**Figure 1:**
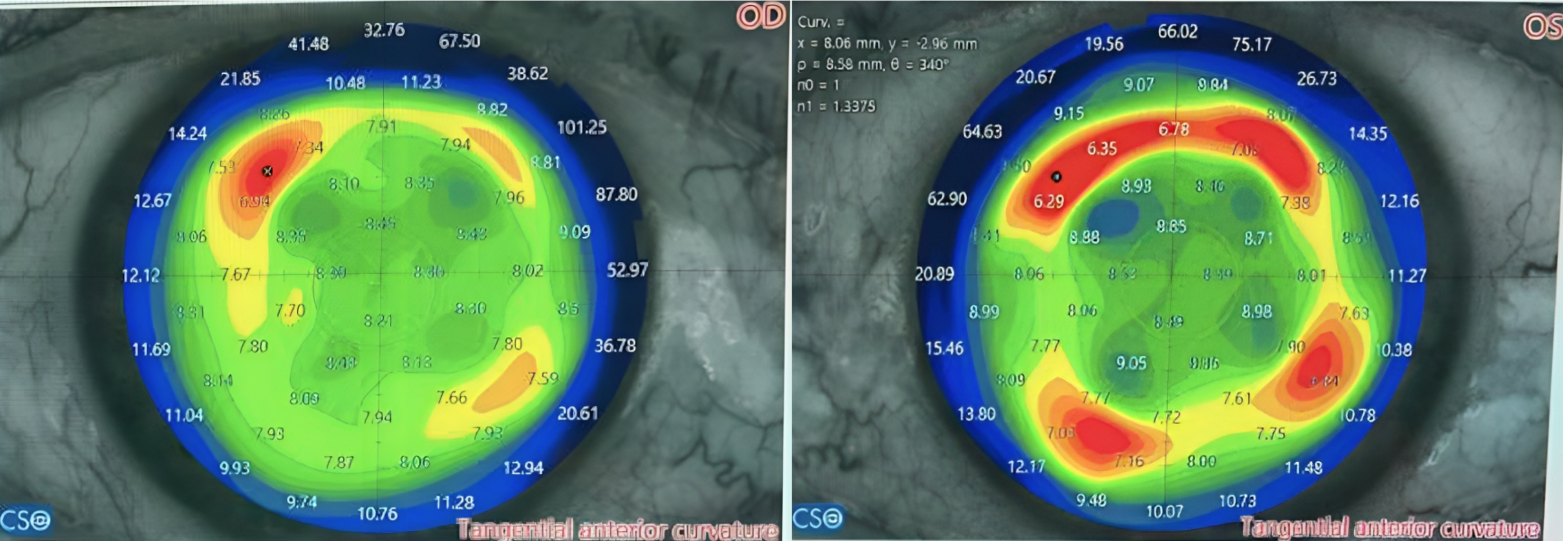
Corneal topography map of both eyes showing irregular astigmatism

**Figure 2:**
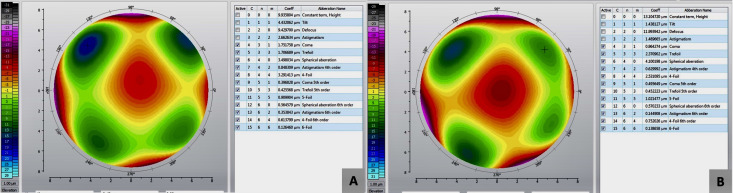
Corneal wavefront analysis of the right eye (A) and left eye (B) depicting increased higher-order aberrations such as coma, spherical aberration, and trefoil aberration

A 69-year-old male patient admitted to our clinic with a complaint of gradually decreased vision both at distance and near in both eyes. He underwent RK elsewhere due to myopia in both eyes 40 years ago. His past and family histories were unremarkable. On examination, the uncorrected distance visual acuity (UDVA) in both eyes was 20/40, and the uncorrected near visual acuity (UNVA) (at 40 cm) was J10. The best-corrected distance visual acuity in the right eye (RE) was 20/25 (+2.00–0.75x80) and in the left eye (LE) was 20/25 (+2.75–0.75x70). Slit-lamp examination was normal except for 4-cut RK scars and mild nuclear sclerosis in both eyes. The intraocular pressure was 14 mmHg in each eye. Corneal topography (Sirius; Costruzione Strumenti Oftalmici, Florence, Italy) revealed flattening in the central region and irregular steepening areas corresponding to RK incisions in both eyes (Figure 1 (Fig. 1)). Corneal wavefront analysis revealed increased higher-order aberrations such as coma, spherical aberration, and trefoil aberration in both eyes (Figure 2A, B (Fig. 2)).

The dilated fundus examination was unremarkable and optical coherence tomography depicted normal foveal contours in both eyes with no evidence of myopic maculopathy. Keratometric measurements were obtained using Zeiss Visuref 150 (Carl Zeiss Meditec AG, Germany), IOL Master 500 (Carl Zeiss Meditec AG, Germany), Topolyzer WaveLight II (Alcon Laboratories, Inc., Fort Worth, ABD), and Sirius topographer^®^. Axial length, anterior chamber depth, and white-to-white distance measurements from IOL Master 500 were used for biometric calculations (Table 1 (Tab. 1)). The internet based IOL power calculator at American Society of Cataract and Refractive Surgery (ASCRS) website (https://www.ascrs.org) served as the instrument for IOL power calculation. Unfortunately, detailed preoperative data for RK was not available. Therefore, the calculation of lens power was conducted using Barrett’s True K formula, with a postoperative target refraction of –0.25 D for the LE. Subsequently, based on the postoperative results of the LE after one week, a target refraction of –0.50 D was chosen for the RE. Alcon Panoptix (Alcon Laboratories, Inc., Fort Worth, USA), a trifocal lens with diffractive properties, was preferred upon the patient’s request for enhanced near vision without the necessity of wearing glasses. Detailed preoperative counseling was provided to the patient regarding the challenges in IOL power calculation after RK, potential refractive errors postoperatively, positive dysphotopsia problems such as halo and glare with diffractive trifocal lenses, and the prolonged period required for the development of refractive stability postoperatively. The patient was informed that the use of EDOF lenses could mitigate potential risks, and that a low-powered pair of glasses might be needed for near vision. However, as the patient unequivocally expressed a preference against the use of reading glasses, a trifocal diffractive lens was recommended. Comprehensive informed consent was obtained.

**Figure 3:**
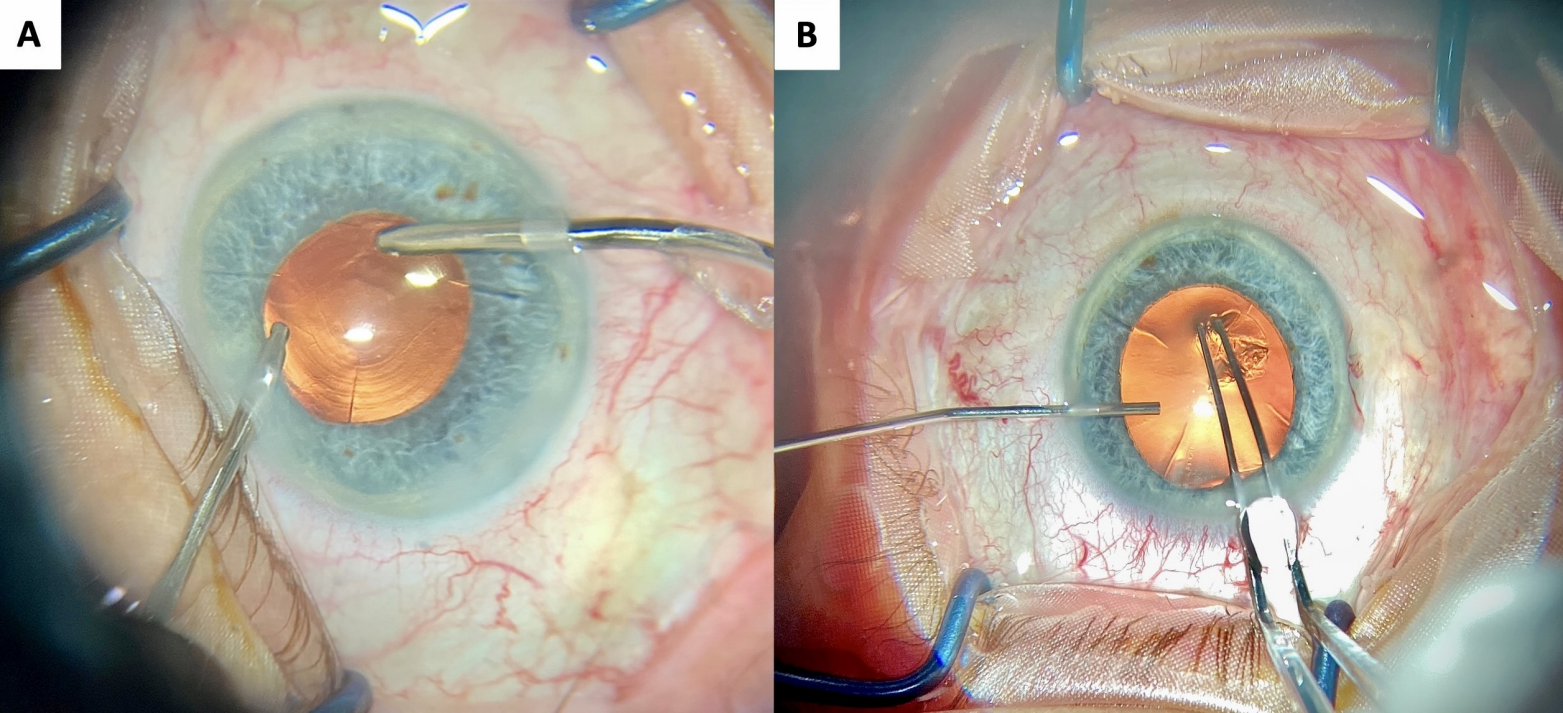
Peroperative picture of the right eye (A) and (B) left eye revealing 4 incisions after radial keratotomy. The corneal incisions are positioned away from the radial keratotomy incisions.

The surgeries for both eyes were performed by the same surgeon (ŞCİ) at a one-week interval. A 2.4 mm keratome was used to create clear corneal incisions, positioned away from the RK incisions (Figure 3A, B (Fig. 3)). Phacoemulsification and intraocular IOL implantation was performed without complications. A 23 D Alcon PanOptix^®^ lens was implanted in the LE, and a 22 D Alcon PanOptix^®^ lens was implanted in the RE. After surgery, the patient was treated with moxifloxacin and dexamethasone eyedrops. Refraction was +1.00–1.25x95 in the RE and +1.25–1.25x90 in the LE in the first month post-surgery. Uncorrected distance visual acuity was 20/32, binocular UDVA was 20/25, and binocular UNVA was J1 in both eyes in the first month. The last follow-up for the assessment of refractive stability was performed six months postoperatively. Refraction was +0.50–1.00x90 in the RE and +0.50–1.25x86 in the LE in the last visit. Binocular was UDVA 20/25 and binocular UNVA was J1 at 6 month. Baseline, 1^st^ month, and 6^th^ month refraction and visual acuity values of the patient are shown in Table 2 (Tab. 2). The patient stated that he no longer needed glasses in his daily life and had no complaints of positive dysphotopsia, such as halo and glare.

## Discussion

Patients who previously underwent RK and desire to be spectacle-free, whether they have refractive errors with clear lenses or cataracts, pose a significant challenge for surgeons. Toric, EDOF, and multifocal IOLs may provide excellent outcomes in selected RK cases that meet certain corneal topographic criteria [5], [7]. Kim et al. [8] reported 20/20 UDVA and J1 UNVA for both eyes of two unilateral refractive lens exchange patients using Oculentis IOLs. Baartman et al. [5] retrospectively reviewed 24 eyes of 12 patients with a history of RK who had undergone phacoemulsification with implantation of the Tecnis Symfony IOL (J&J Vision). They reported that 78% of patients reported satisfaction with their vision after surgery and 44% of patients reported being spectacle-free for all tasks [5].

Martín-Escuer et al. [9] retrospectively examined 17 eyes from nine consecutive patients who underwent cataract or refractive lens exchange surgery involving multifocal IOL implantation and who had also previously undergone RK surgery. They employed the double-K formula to calculate the power of the IOL the target was fixed at –1.00 D in all cases. They stated that their study at 6 months post-surgery revealed no significant change compared to before the surgery both in UDVA and distance-corrected visual acuity (DCVA) (p>0.1). Mean values post-surgery were about 20/63 and 20/32 for UDVA and DCVA, respectively. Only two eyes (11.76%) achieved a UDVA of 20/25 or better. DCVA revealed similar values between pre- and post-surgery but being more eyes before the surgery with a DCVA of 20/20 or better. A better outcome was found for distance-corrected near visual acuity (about 20/25), which was equal to or better than 20/20 for five eyes [9]. Contrary, previous studies by Gupta et al. [10], Kim et al. [8], and Nuzzi et al. [4] involving the implantation of different multifocal IOL models reported better outcomes than Martín-Escuer et al. [9]. Case reports involving patients with RK who underwent multifocal IOL implantations in the literature are summarized in Table 3 (Tab. 3) [4], [8], [10], [11]. 

Radial keratotomy leads to the peripheral elevation and central flattening of the cornea, impacting both the anterior and posterior radius of curvature similarly [12]. In the present study, the corneal topography map revealed both central flattening and peripheral localized steepening as well. The deep corneal marks of RK contribute to a biomechanically unstable cornea, leading to irregular astigmatism, vision fluctuations during the day, as well as glare and halos [13]. The RK eyes have the difficulty in determining the true corneal refractive power. It has been suggested that delayed healing of incisional wounds in RK could contribute as a partial mechanism for the hyperopia observed after cataract surgery [14]. The ASCRS calculator, designed for eyes with a history of refractive surgery, facilitates the precise determination of IOL power for individuals with prior RK [15].

There is no perfect formula for IOL power calculation for patients with prior corneal refractive surgery and the accuracy of each formula remains controversial [16]. Barret’s True K formula is derived from the Barrett Universal II formula, which is specifically designed for eyes that have undergone prior corneal refractive surgery [1], [17]. Turnbull et al. [18] retrospectively evaluated medical records of a total of 52 eyes (34 patients) who had sequential RK and cataract surgery. They examined 7 IOL calculation formulae: True K [History], True K [Partial History], True K [No 44 History], Double-K Holladay 1 (DK-Holladay-IOLM), Potvin-Hill, Haigis and Haigis with a –0.50 D offset. They stated that best results were achieved with the True K [History] [18]. In another study, Soare et al. [3] retrospectively examined 100 eyes (65 patients) with previous RK who had undergone routine cataract surgery with a monofocal IOL and stated that standard Haigis formula aiming for emmetropia achieves better IOL power estimation compared with the traditional Double-K SRK/T, with 73.7% of eyes within 1.0 D in eyes with prior RK. They also pointed out that it is a reliable and simple method when refractive history is not available [3].

In the present case, he has significant hyperopia and HOAs. Despite concerns about the potential increase in HOAs following multifocal IOL implantation post-refractive surgery, a trifocal diffractive lens was recommended because the patient is seeking spectacle independence and enhanced near vision. Although irregular astigmatism is common among RK patients, and our patient has topographic irregularities, we did not prefer a toric IOL because of the relatively low preoperative astigmatism values.

## Conclusion

While the implantation of monofocal IOLs is commonly considered safer for patients with uncertain surgical outcomes, multifocal IOLs may also have advantages for those who have undergone RK and wish to enhance near vision. A comprehensive examination of preoperative topography is crucial in RK patients and treatment for each patient should be customized.

## Notes

### Authors’ ORCIDs

- Şefik Can İpek: 0000-0002-4651-8177
- Seher Köksaldı: 0000-0001-8235-4088

### Statement of informed consent

Written informed consent was obtained from the patient to include the ocular imaging, genetic testing results, and clinical data in the publication.

### Competing interests

The authors declare that they have no competing interests.

## Figures and Tables

**Table 1:**
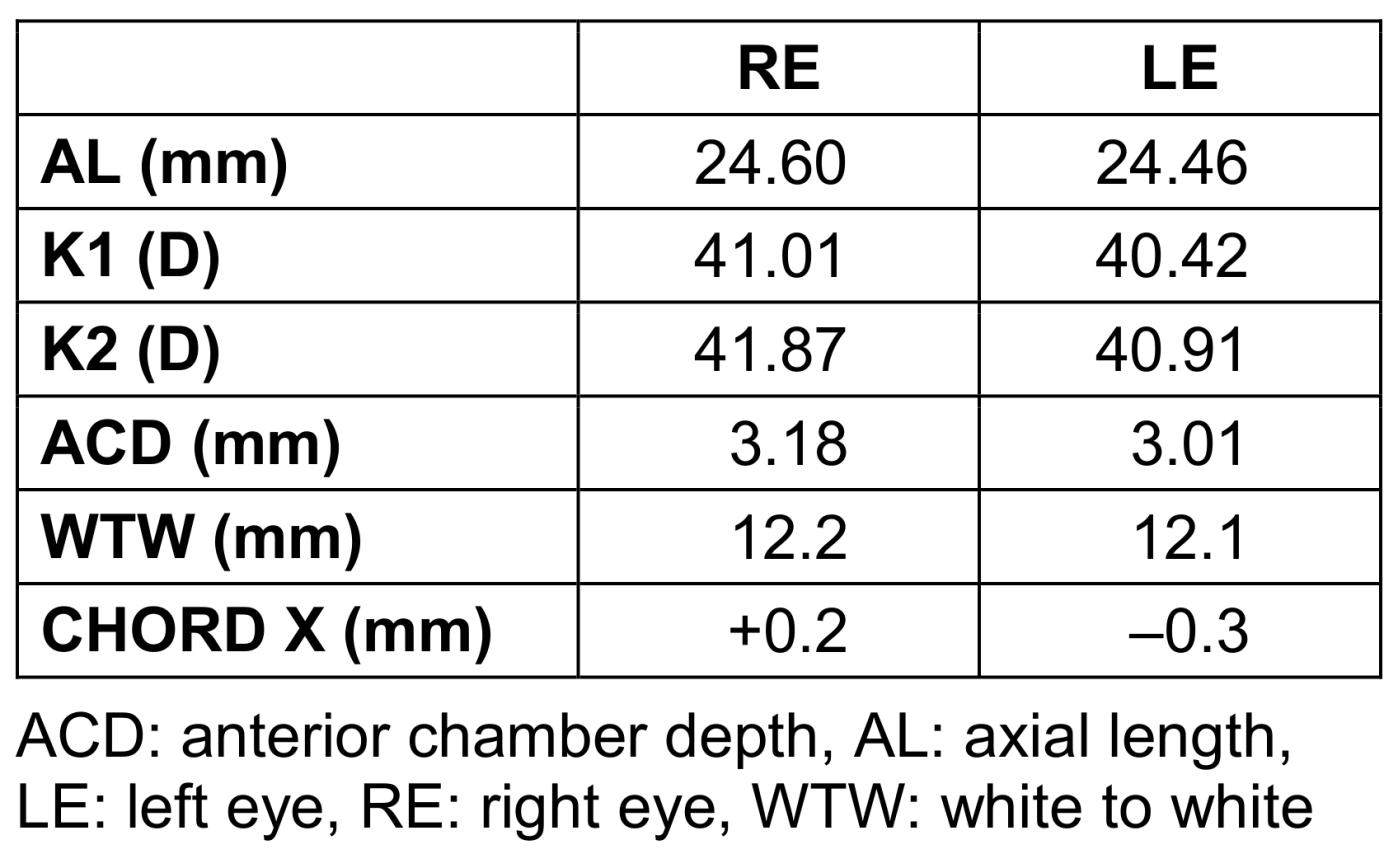
Axial length, anterior chamber depth, and white-white distance measured with IOL Master 500

**Table 2:**
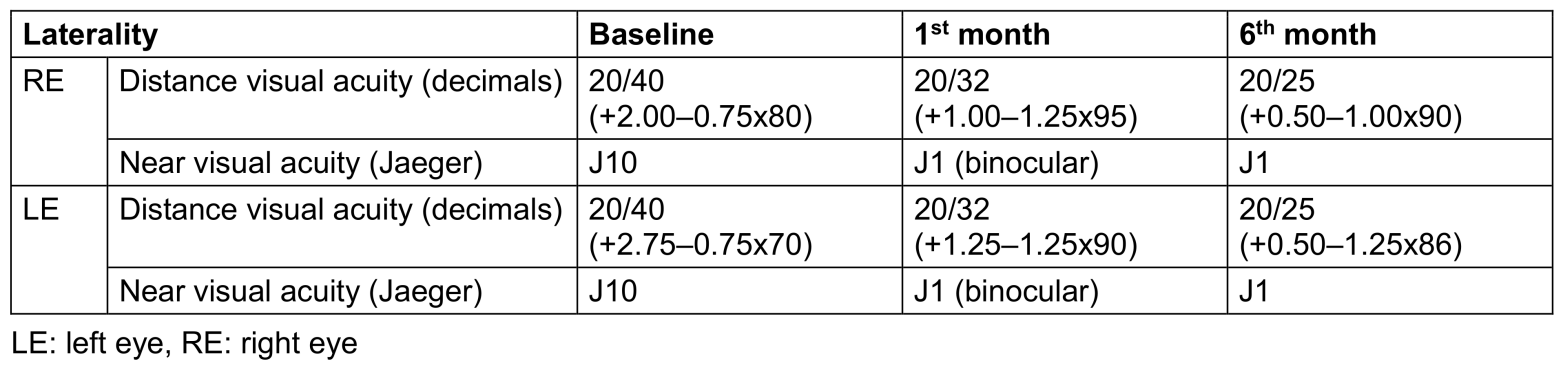
Baseline, 1^st^ month, and 6^th^ month refraction and visual outcomes of the present case

**Table 3:**
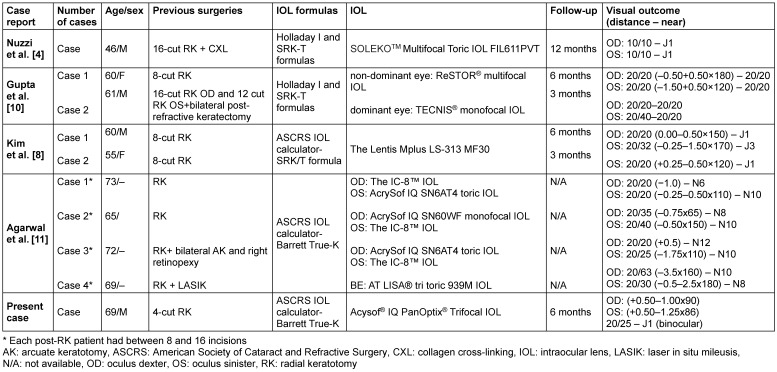
Case reports involving patients with RK who underwent multifocal IOL implantations in the literature
